# Transformation of HHV‐8‐negative idiopathic multicentric Castleman disease into diffuse large B‐cell lymphoma: A case report from Nepal

**DOI:** 10.1002/ccr3.7903

**Published:** 2023-09-11

**Authors:** Ashwini Gupta, Ayush Anand, Salman Haidar Hussain, Raju Shah, Rajesh Ranjan, Viplaw Subedi, Aastha Baral, Akshat Mishra, Anju Pradhan, Soniya Dulal

**Affiliations:** ^1^ BP Koirala Institute of Health Sciences Dharan Nepal; ^2^ Department of Internal Medicine BP Koirala Institute of Health Sciences Dharan Nepal; ^3^ Department of Pathology BP Koirala Institute of Health Sciences Dharan Nepal

**Keywords:** case report, Castleman disease, idiopathic, multicentric, non‐Hodgkin lymphoma

## Abstract

**Key Clinical Message:**

Idiopathic Castleman disease transforming into Diffuse Large B‐cell Lymphoma has an aggressive course and can lead to mortality. Hence, early diagnosis and intervention are required.

**Abstract:**

Idiopathic Castleman disease transforming into non‐Hodgkin lymphoma has an aggressive course, poor prognosis, and high mortality rate. Hence, early diagnosis and intervention are necessary. In a developing country like Nepal, where infectious diseases, particularly TB, are high, concomitant infection worsens the disease course. It also poses a diagnostic challenge as the clinical presentation may be similar.

## Graphical abstract

Microscopic and Immunohistochemistry findings in Castleman Disease.

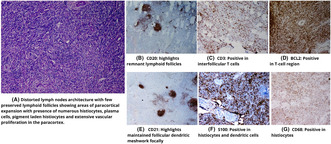

## 1 INTRODUCTION

Castleman disease (CD), also known as angiofollicular lymph node hyperplasia, represents a class of lymphoproliferative disorders with characteristic histopathological features of hyaline type, plasma cell type, or mixed type on biopsy^1^, ^2^ and can be divided into unicentric and multicentric CD.^3^ In the context of low‐middle‐income countries, the data regarding the incidence of CD is limited.^4^ Unicentric CD (UCD), accounting for about three‐fourths of the total CD cases,^4^ represents the involvement of a single lymph node and is usually a reversible condition with localized and mild symptoms.^2^, ^5^, ^6^ Multicentric CD (MCD) involves multiple lymph nodes with mild to severe presentations, accounting for about 23% of all CD cases.^4^, ^5^ MCD can be further classified into HHV‐8‐associated MCD, idiopathic MCD (iMCD), and POEMS‐associated MCD.^1^ Idiopathic multicentric Castleman disease constitutes the majority of MCD cases and has a poor prognosis.^6^, ^7^ The patients can present with fever, night sweats, weight loss, hepatomegaly, splenomegaly, and ascites.^7^ Laboratory investigations can show elevated ESR, CRP, anemia, thrombocytopenia, hypoalbuminemia, positive ANA test, and elevated IL‐6.^7^ Detailed history and laboratory workup are required to diagnose iMCD based on consensus diagnostic criteria.^3^ These patients can be divided into non‐severe and severe categories for management purposes.^6^ For non‐severe patients, anti‐IL‐6 monoclonal antibody therapy with siltuximab (11 mg/kg every 3 weeks) is the first‐line pharmacological modality.^6^ Alternatively, rituximab can also be used with steroids as adjunctive therapy for disease control.^5^, ^6^ In severe patients, combination chemotherapy of the R‐CHOP regimen (rituximab‐cyclophosphamide, doxorubicin, vincristine, and prednisone) should be employed.^5^ Multicentric CD can be associated with various solid tumors and hematological malignancies.^7^ It may transform into non‐Hodgkin lymphoma (NHL), leading to mortality.^7^, ^8^ Herein, we present the case of a 55‐year‐old Asian male with HHV‐8‐negative iMCD managed with combination chemotherapy, which transformed into NHL with tuberculosis, leading to the patient's death.

## 2 CASE REPORT

**FIGURE 1:**
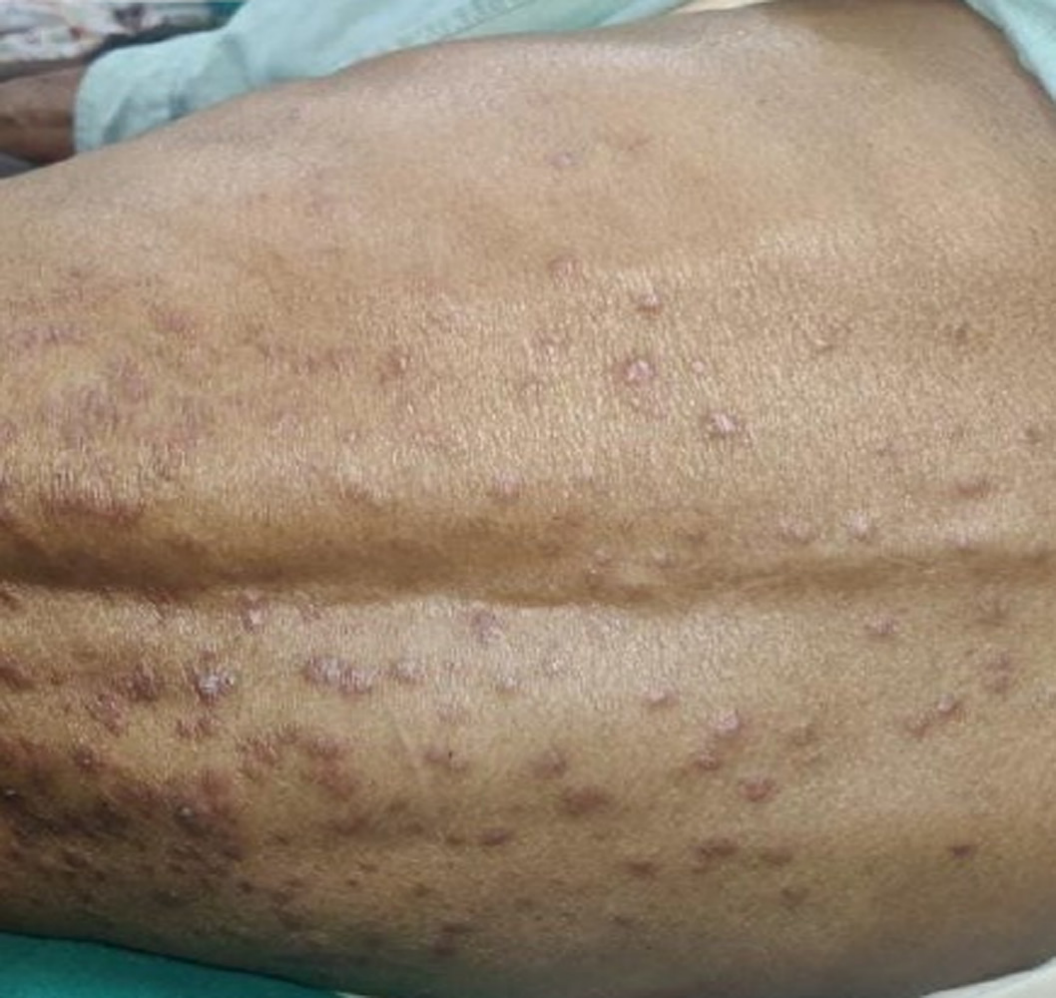
Cherry red hemangioma on back of the patient.

**FIGURE 2:**
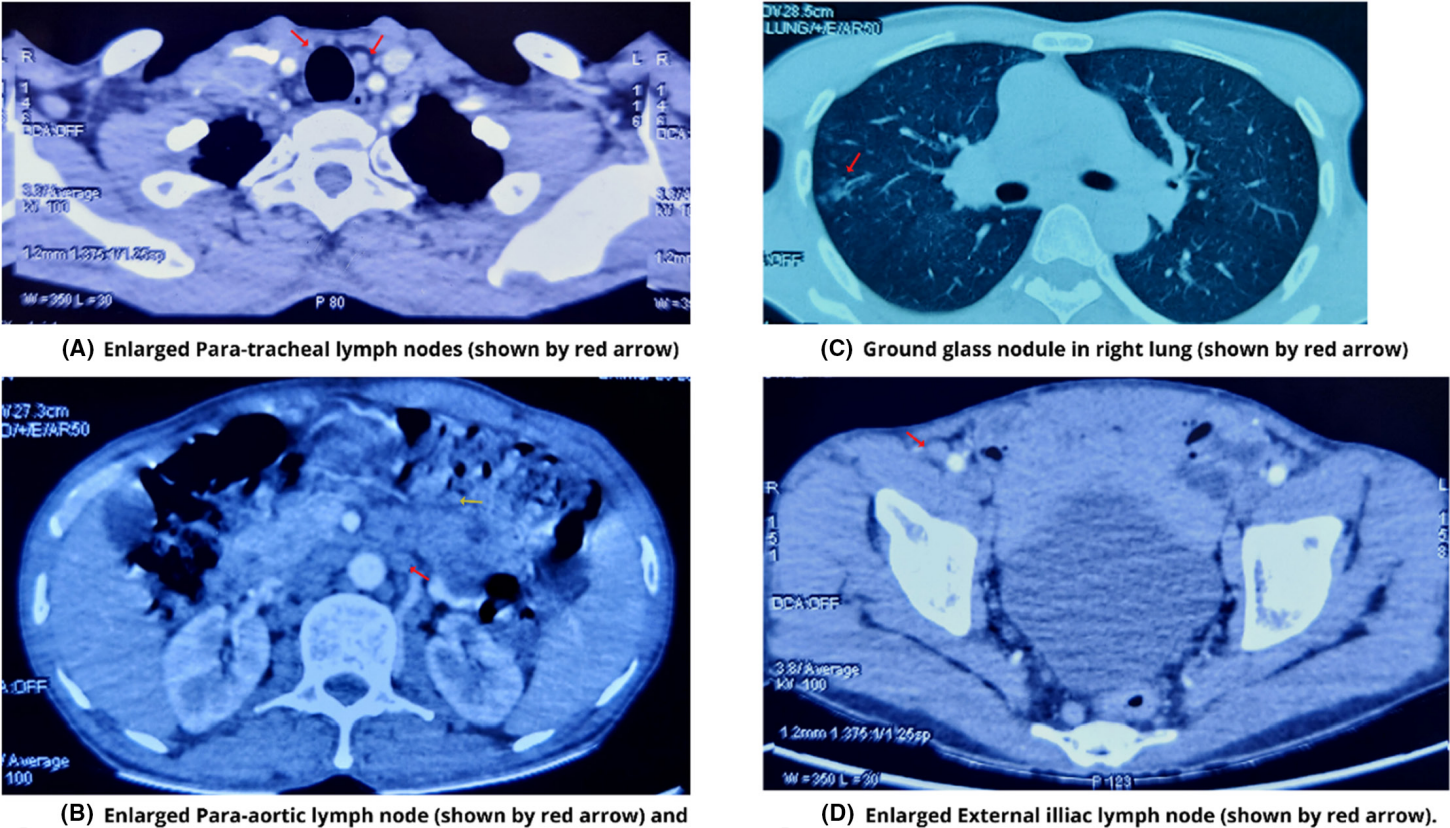
Computed tomography scan of the neck, chest, abdomen, and pelvis. (A) Enlarged Para‐tracheal lymph nodes (shown by red arrow). (B) Enlarged para‐aortic lymph node (shown by red arrow) and peri‐pancreatic lymph node (shown by yellow arrow). (C) Ground glass nodule in right lung (shown by red arrow). (D) Enlarged external iliac lymph node (shown by red arrow).

**FIGURE 3:**
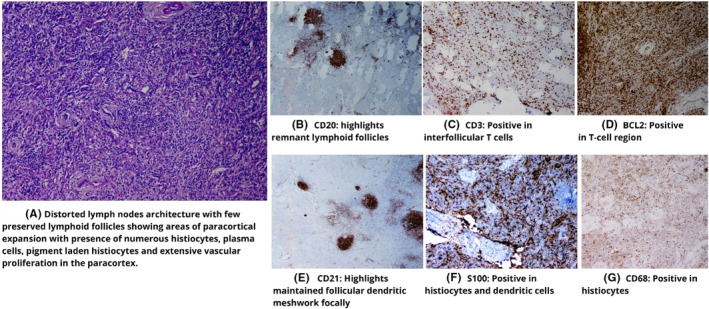
Immunochemistry findings of the patient. (A) Distorted lymph nodes architecture with few preserved lymphoid follicles showing areas of paracortical expansion with presence of numerous histiocytes, plasma cells, pigment laden histiocytes, and extensive vascular proliferation in the paracortex. (B) CD20: highlights remnant lymphoid follicles. (C) CD3: positive in interfollicular T cells. (D) BCL2: positive in T‐cell region. (E) CD21: highlights maintained follicular dendritic meshwork focally. (F) S100: positive in histiocytes and dendritic cells. (G) CD68: positive in histiocytes.

**FIGURE 4:**
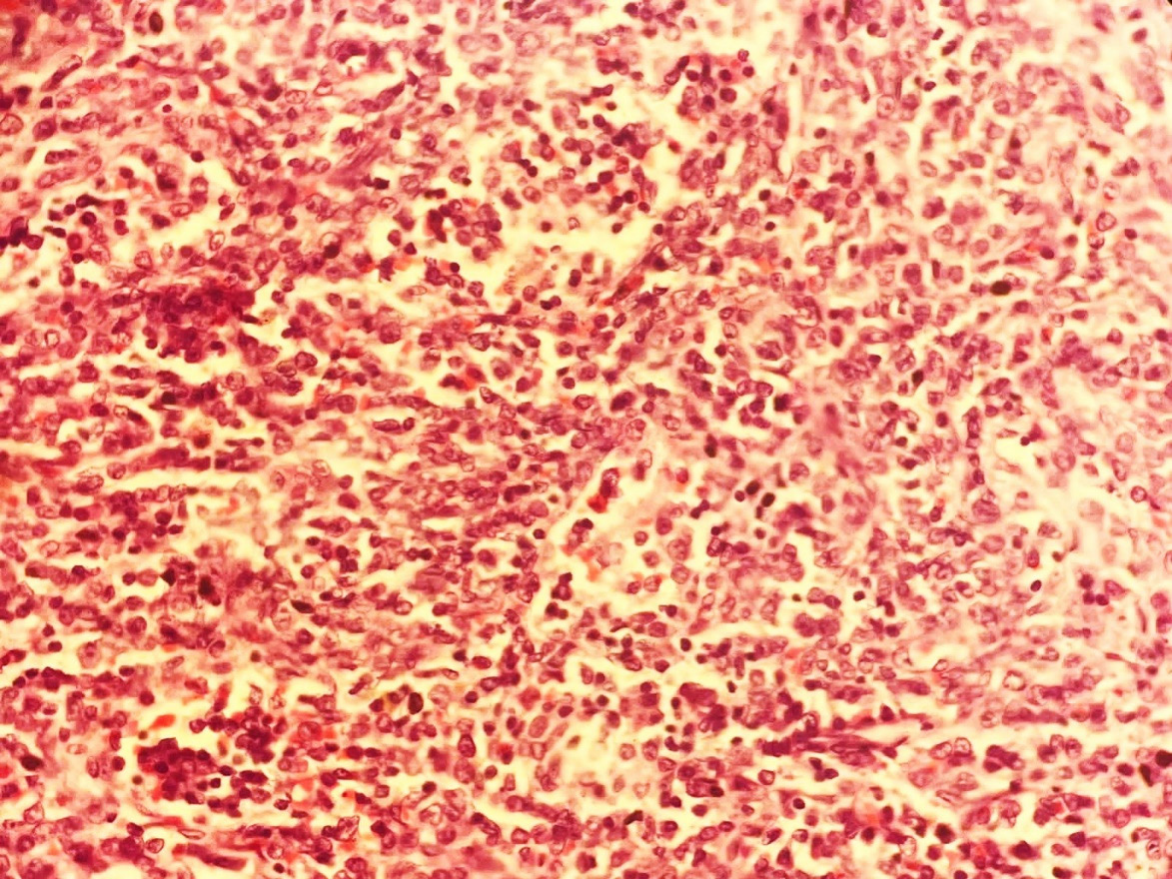
Histopathology showing diffuse proliferation of lymphoid cells with centroblasts. Few cells show nuclear irregularity with prominent nucleoli.

A 55‐year‐old Asian male presented in August 2021 with fever, abnormal sensation in the abdomen, fatigue, and anorexia for 1 month. At arrival, he was tachycardic, tachypneic, febrile, and ill‐appearing. There were cherry red hemangiomas present over the trunk (Figure 1). He had pallor and generalized lymphadenopathy with clear lung fields and no cardiac murmur. The patient was advised for various laboratory and imaging investigations. Initial laboratory studies (Table 1) revealed anemia, leucopenia, neutrophilia, hypoalbuminemia, decreased UIBC, hyponatremia, hypokalemia, hypochloremia, elevated serum creatinine, and elevated ESR and CRP. Serology investigations revealed ANA‐positive status. Further, the ANA profile was not done due to financial reasons. Over time, the patient developed fever, and a fever workup was done where all samples had no growth except for the urine with growth of Proteus mirabilis, suggesting urinary tract infection. Echocardiography showed moderate aortic and tricuspid regurgitation. A computed tomography scan of the neck, chest, abdomen, and pelvis showed: enlarged few bilateral submandibular and upper cervical lymph nodes (the largest measuring 11 × 7 mm), multiple prevascular, paratracheal, and subaortic lymph nodes (some conglomerated and largest measuring 26.3 × 13.3 mm), multiple enlarged mesenteric, periportal, peripancreatic, paraaortic, aortocaval, common, and external iliac lymph nodes (the largest 33.7 × 19.8 mm, most of them conglomerated), splenomegaly (15.3 cm), and few ground glass nodules in both lungs. A few of the CT findings are shown in Figure 2. Figure 3 and Table 2 show the immunohistochemistry study findings. The lymph node biopsy revealed a combination of hypervascularity and plasmacytic features, suggesting a mixed type of CD. Various infectious causes, such as HHV‐8, EBV infection, and HIV, were considered differentials. Other differentials were malignant/lymphoproliferative disorders, such as lymphoma, multiple myeloma, and POEMS syndrome. Considering the financial aspects of the patient, limited investigations were done that too free of cost, on hospital request. Hence, detailed immunohistochemistry and fluorescence in situ hybridization tests were not done. Based on the clinical evaluation, the patient was diagnosed with iMCD with HHV‐8‐negative status. The patient underwent six cycles of the CHOP regimen (Table 3). During six cycles of CHOP, the patient's condition improved. However, on follow‐up in December 2022, 16 months after the initial presentation, the patient's condition worsened with symptoms of fever and cervical lymphadenopathy, for which we sent a lymph node biopsy. The left upper cervical region biopsy suggested a centroblastic variant diffuse large B‐cell non‐Hodgkin's lymphoma (Figure 4). In the line of management for the lymph node, the AFB examination showed positive, and tuberculosis (TB) was diagnosed. With ECOG performance status of 3, chemotherapy was halted, and treatment for TB was started. Unfortunately, the patient died in the same month due to untreated TB and coexisting comorbidities.

**TABLE 1:** Laboratory investigations of the patient.

Investigations	Results	Reference range
Hb (g/dL)	6.9	11–16
TLC (cells/mm^3^)	700	4000–11,000
DLC	N85 L10 M05 B0 E0	N40‐75 L20‐45 M0‐10 B0‐2 E 0‐4
Platelets (cells/mm^3^)	155,000	150,000–450,000
PT (s)	14	12–16
INR	1.0	
MCV (fL)	83.2	80–96
MCH (pg)	25.4	27–34
MCHC (g/dL)	30.6	32–36
RBC (million/mL)	2.91	1.5–4.5
Serum iron (μg/dL)	64	33–193
UIBC (μg/dL)	101	125–345
Serum creatinine (mg/dL)	1.4	0.6–1.3
Serum urea (mg/dL)	45	10–50
Serum sodium (mmol/L)	128	135–150
Serum potassium (mmol/L)	3.8	3.5–5.1
Serum chloride	95	98–107
ESR (mm/First hour)	57	0–9
CRP (mg/L)	192	0–5
ANA (AU/mL)	48.93	Negative < 40; Positive > 40
Total protein (g/dL)	7.1	6.0–8.3
Albumin (g/dL)	3.0	3.5–5.0
Total bilirubin (mg/dL)	0.9	0.2–1.2
ALT (U/L)	34	09–43
AST (U/L)	20	10–35
ALP (U/L)	67	35–130
C3	92	90–180
C4	35	10–40
HIV, HBsAg	Negative	
HCV	Negative	

Abbreviations: ALP, alkaline phosphatase; ALT, alanine transaminase; ANA, antinuclear antibody; AST, aspartate transaminase; B, basophil; CRP, C‐reactive protein; DLC, differential leucocyte count; E, eosinophil; ESR, erythrocyte sedimentation rate; Hb, hemoglobin; HBsAg, Hepatitis B surface antigen; HCV, hepatitis C virus; HIV, human immunodeficiency virus; INR, international normalized ratio; L, lymphocytes; M, Monocyte; MCH, mean corpuscular hemoglobin; MCHC, mean corpuscular hemoglobin concentration; MCV, mean corpuscular volume; N, neutrophil; PT, prothrombin time; RBC, red blood cell; TLC, total leucocyte count; UIBC, universal iron binding capacity.

**TABLE 2:** Immunohistochemistry study findings in the patient.

Immunohistochemistry studies	Findings
CD20 and PAX 5	Highlights remnant lymphoid follicles
CD3	Positive in interfollicular T cells
CD30	Negative
BCL2	Positive in the T‐cell region
Ki67	10%–15%
CD21	Highlights maintained follicular dendritic meshwork focally
S100	Positive in histiocytes and dendritic cells
CK	Negative
CD68	Positive in histiocytes
CD1A	Negative
CD34	Highlights vascular proliferation
EBV, CD15, and Melan A	Negative

**TABLE 3:** Laboratory investigations before each cycle of chemotherapy.

Investigation	05/01 (PC1)	05/24 (PC2)	06/16 (PC3)	07/06 (PC4)	07/26 (PC 5)	08/16 (PC 6)	Reference range
Hemoglobin (gm/dl)	8.2	8.3	7.6	7.8	9.7	9.5	11–16
TLC (cell/mm^3^)	5600	6910	8500	8200	4200	4600	4000–11,000
DLC (%)	N 69 L 18	N 73 L 23	N 70 L 20	N 71 L 21	N 38 L 49	N 56 L 24	N 40–75 L 20–45
Platelet count (cell/mm^3^)	258,000	195,000	123,000	133,000	170,000	183,000	150,000
Serum urea (mg/dL)	28	48.5	33.2	35	20.69	37	10–50
Serum creatinine (mg/dL)	1.3	1.2	0.9	1.1	1.23	1.34	0.6–1.3
Sodium (mmol/L)	138	140	140	139	138.7	141	136–145
Potassium (mmol/L)	3.2	4.8	4.7	4.5	4.0	4.2	3.5–5.0

Abbreviations: DLC, differential leucocyte count; L, lymphocyte percentage; N, neutrophil percentage; TLC, total leucocyte count.

## 3 DISCUSSION

Idiopathic multicentric Castleman disease accounts for about one‐third to two‐thirds of all MCD cases.^7^ Early diagnosis and intervention are required as the 5‐year survival rate ranges from 55% to 77%, which is very low.^6^ In our case, the patient defaulted due to financial reasons. This reflects that financial constraints in low‐middle‐income countries can act as barriers to healthcare, leading to delays in diagnosis and treatment and increased mortality. Idiopathic multicentric Castleman disease can be diagnosed based on the Castleman Disease Collaborative Network (CDCN) consensus diagnostic criteria.^3^ Similar to Fajgenbaum et al., cherry red hemangioma was seen in our patient, which is very rare finding.^9^ A systematic review by Liu et al.^7^ found that all the cases of iMCD had multicentric lymphadenopathy, and fever was reported in 26% of the cases. Moreover, hepatomegaly or splenomegaly was reported in 40% of cases on CT scans.^7^ Decreased hemoglobin was found in 62%, elevated ESR in 34%, elevated CRP in 51%, hypoalbuminemia in 45%, and positive ANA test in 12% of the reported cases.^7^ Similar to these findings, our patient had a fever and multicentric lymphadenopathy. The patient also had low hemoglobin, elevated ESR, elevated CRP, hypoalbuminemia, and ANA‐positive status. Moreover, the patient had splenomegaly, contributing to a dragging sensation in the abdomen and anorexia. One interesting finding was the presence of leucopenia, which has not been reported in iMCD cases. The lymph node biopsy revealed characteristic histopathologic findings suggestive of mixed‐type CD.^3^ Also, a CT scan revealed multiple lymphadenopathies. Hence, our patient fulfilled the major and minor diagnostic criteria of iMCD by CDCN.^3^ Other infectious causes which mimic iMCD, such as HHV‐8, EBV infection, and HIV, were excluded. Based on clinical evaluation and investigations, we excluded other malignant/lymphoproliferative disorders, such as lymphoma, multiple myeloma, and POEMS syndrome. So, after addressing all the major, minor, and exclusion criteria, a diagnosis of HHV‐8‐negative iMCD was made.

Various studies have shown that iMCD patients benefit from the CHOP regimen, and most patients remain alive on follow‐up at 3 years.^10^, ^11^, ^12^, ^13^ These patients usually require 1–4 cycles of combination chemotherapy.^10^ In our case, the patient was managed with six cycles of the CHOP regimen. The patient showed improvement and was asymptomatic till 2 months after the sixth cycle. Since the patient's condition worsened, we did a lymph node biopsy, which suggested DLBCL. A review by Larroche et al. pointed toward an increased incidence of NHL in MCD cases.^8^ A systematic review revealed that six out of 128 cases of MCD had NHL, of which two were DLBCL.^7^ This suggested that patients with iMCD can transform to NHL. Also, malignancies with MCD significantly increase the risk of death.^7^ Larroche et al.^8^ also found increased mortality in MCD cases. Along with AFB‐positive tuberculosis, poor health status and an aggressive course of the tumor might have led to the death of our patient. The presence of TB may sometimes lead to diagnostic dilemmas as the presentation of TB can be similar to iMCD and NHL.

Our case is unique as it is the first case of iMCD transforming to NHL reported in Nepal. Diagnosing and treating these cases are incredibly challenging in low‐resource settings and patients of low socioeconomic status. In our case, we could not manage the patient with IL‐6 inhibitors, the first‐line therapy for managing iMCD. This was because of less availability of drugs and the patient's financial status, which did not allow the procurement of costly medications. These issues need to be addressed by stakeholders. The government should launch a roadmap for easy procurement of chemotherapeutic drugs and financial aid schemes for timely and effective management of cases.

## 4 CONCLUSION

Idiopathic multicentric Castleman disease transforming into NHL can rapidly lead to mortality. Hence, early diagnosis and intervention are required. In low‐resource settings, where the prevalence of infectious disease, particularly TB, is high, concomitant infection worsens the disease course. It even leads to confusion in diagnosis because of overlapping constitutional symptoms like fever, night sweats, lymphadenopathy, and anorexia.

## AUTHOR CONTRIBUTIONS

**Ashwini Gupta:** Conceptualization; data curation; project administration; supervision; validation; visualization; writing – original draft; writing – review and editing. **Ayush Anand:** Conceptualization; data curation; project administration; supervision; validation; visualization; writing – original draft; writing – review and editing. **Salman Haidar Husain:** Data curation; writing – original draft; writing – review and editing. **Raju Shah:** Data curation; writing – original draft; writing – review and editing. **Rajesh Ranjan:** Data curation; writing – original draft; writing – review and editing. **Viplaw Subedi:** Data curation; writing – original draft; writing – review and editing. **Aastha Baral:** Data curation; writing – original draft; writing – review and editing. **Akshat Mishra:** Conceptualization; data curation; project administration; supervision; validation; visualization; writing – review and editing. **Anju Pradhan:** Conceptualization; data curation; project administration; supervision; validation; visualization; writing – original draft; writing – review and editing. **Soniya Dulal:** Conceptualization; data curation; project administration; resources; supervision; validation; visualization; writing – review and editing.

## FUNDING INFORMATION

The authors did not receive any funding for this manuscript.

## CONFLICT OF INTEREST STATEMENT

The authors have no conflict of interest to declare.

## ETHICS STATEMENT

Ethical approval was not required for this case report.

## CONSENT

Written informed consent was obtained from the patient to publish this report in accordance with the journal's patient consent policy.

## Data Availability

All data pertaining to this case is made available within the manuscript.
